# Physeal bar resection by modified arthroscopically assisted surgery in a closed osteocavity

**DOI:** 10.3389/fped.2023.1157192

**Published:** 2023-10-17

**Authors:** Han Xiao, Miao Li, Qian Tan, Weihua Ye, Jiangyan Wu, Haibo Mei, Guanghui Zhu, An Yan

**Affiliations:** ^1^ Department of Pediatric Orthopedics, Hunan Children’s Hospital, Changsha, China; ^2^ The School of Pediatrics, Hengyang Medical School, University of South China, Changsha, China; ^3^ Hunan Provincial Key Laboratory of Pediatric Orthopedics, Hunan Children's Hospital, Hunan, China

**Keywords:** physeal bar, arthroscopy, all-inside, closed osteocavity, direct visualization

## Abstract

**Background:**

Physeal bar resection has been used for partial growth arrest treatment for a decade while removing the bony bar minimally invasively and accurately is challenging. This research aims to illustrate a modified arthroscopically assisted surgery, by which all the procedure was under all-inside visualization, without the constant exchange between burring under fluoroscopy, followed by irrigation, suction, and arthroscopy of the canal.

**Methods:**

We retrospectively reviewed the patients who sustained physeal bar resection under direct all-inside visualization of the arthroscope during 2016–2021. Patients who underwent physeal bar resection with the aid of an arthroscope for identifying the physeal cartilage but not resecting and visualizing the physeal bar simultaneously were excluded from this study.

**Results:**

In total, nine patients with ten related joints were included in this study. All the patients were followed up for at least two years. The average following time was 28.5 ± 6.7 months. Eight patients with nine related joints had an improvement of angular deformity, averaging 8.3 ± 6.9 degrees, and one had a worsening of the angular deformity. All the patients had a leg length discrepancy improvement, while four patients still had LLD >1 cm. The surgery time was 3.1 ± 0.7 h. There were no postoperative fractures, infections, or intraoperative complications such as neurovascular injury.

**Conclusions:**

Using clamps to form a closed osteocavity could make physeal bar resection under all-inside arthroscopic visualization feasible, which is minimally invasive, accurate, and safe.

## Graphical Abstract

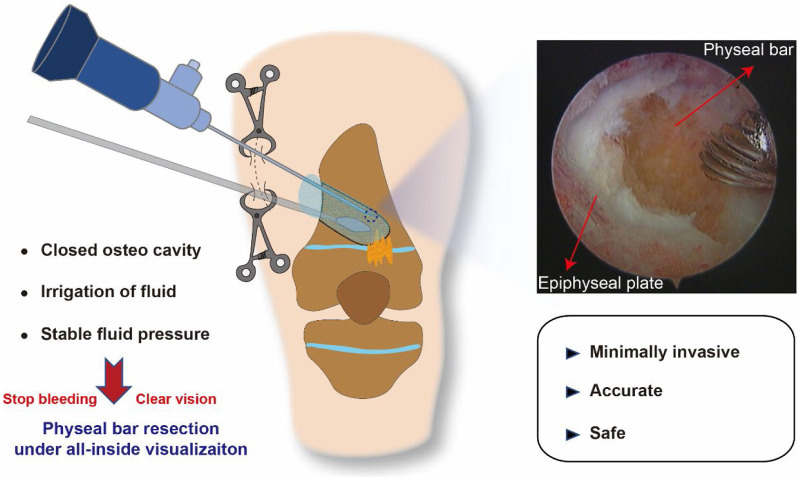

## Introduction

Physeal bar is an uncommon but well-recognized complication secondary to epiphyseal fracture, infection, tumor, irradiation, or iatrogenic insertion of metal across the epiphysis (1). Depending on the bar size, location, and the remaining epiphyseal growth potential, the physeal bar may cause progressively angular and longitudinal deformity (2, 3). Physeal bar resection has been reported as an accepted way to restore the growth ability and release the bar's tethering effect (4, 5). Traditional indications for physeal bar excision include growth plate arrest <50% of the involved physis, and at least two years of growth remaining at the involved physis (3). However, the most significant problem encountered during the resection of a physeal bar is difficulty in visualizing the bar and the surrounding normal physis. Avoiding excessive resection of physeal cartilage is challenging, especially for the central or mixed-type physeal bar.

Many techniques have been reported to remove the physeal bar. The conventional surgical approach is through a metaphyseal window or tunnel with the aid of a dental mirror for visualization (6). In 1992, Striker first reported the physeal bar resection using an arthroscope (7). However, the surgeon can't burr and use the mirror or arthroscope simultaneously due to the limited tunnel space and bloody vision, which inevitably damages the physis cartilage. When angular correction is needed simultaneously, some surgeons apply complete transverse osteotomy of the metaphysis near the bar with a direct vertical approach, which could allow visualization of a centrally located bar and the adjacent healthy physis (8, 9). Still, this procedure is more invasive, and the indications for corrective osteotomy during bridge resection are controversial (10). Recently, the navigation system has been widely used in orthopedic surgery and also has been introduced to physeal bar resection. At the same time, the surgeon can't remove the physeal bar under direct visualization, and an arthroscope is still needed to identify the cartilage ring in this procedure (11, 12). A more accurate, minimally invasive, and efficient method to remove the physeal bar still needs to be explored.

In this study, we modified the traditional arthroscopic surgery and temporally sutured the skin incision to create a closed osteocavity. With this new technique, the surgeon can remove the physeal bar under all-inside arthroscopic visualization. It made the surgery minimally invasive, accurate, and safe, significantly lowering the threshold for using arthroscopy to remove the bone bridge. This study aimed to illustrate and evaluate this minimally invasive procedure.

## Patients and methods

### Patients

After institutional review board approval (KYSQ2021-017), we retrospectively reviewed the patients who underwent physeal bar resection under the direct all-inside visualization of the arthroscope during 2016–2021. Patients who underwent physeal bar resection with the aid of an arthroscope for identifying the physeal cartilage but not resecting and visualizing the physeal bar simultaneously were excluded from this study. Physeal growth arrest was diagnosed by clinical and tomography evidence. The indications for surgery were as follows: no more than 50% of physeal closure and more than two years or 2 cm of skeletal growth remaining based on predicted bone age and menstrual history (10). The indication for epiphysiodesis is based on the senior surgeon's experience. Consent for publication was obtained from the legal guardians of the patients.

### Preoperative planning

A modified mapping method was used to calculate the size of a physeal bar by CT data (Supplementary Figure S1) (13, 14). Standard entire lower limb radiographs were taken in the anteroposterior and lateral views to assess deformity and shortening of the lower limb. The physeal bar was identified by the C.T. and x-ray. Then, the potential tunnel used for physeal resection was designed, and the angle and length were calculated. The tunnel is created based on the location, shape, and size of the physeal bar, which is like a cylinder. It must simultaneously facilitate arthroscope lens placement without affecting the burr operation. When the physeal bar is located in the medial of the bone's midline, the tunnel entrance is lateral, and vice versa. Generally, the angle between the tunnel and the long axis of the tibia or femur should be more than 30°.

### Surgical technique

**Figure 1:**
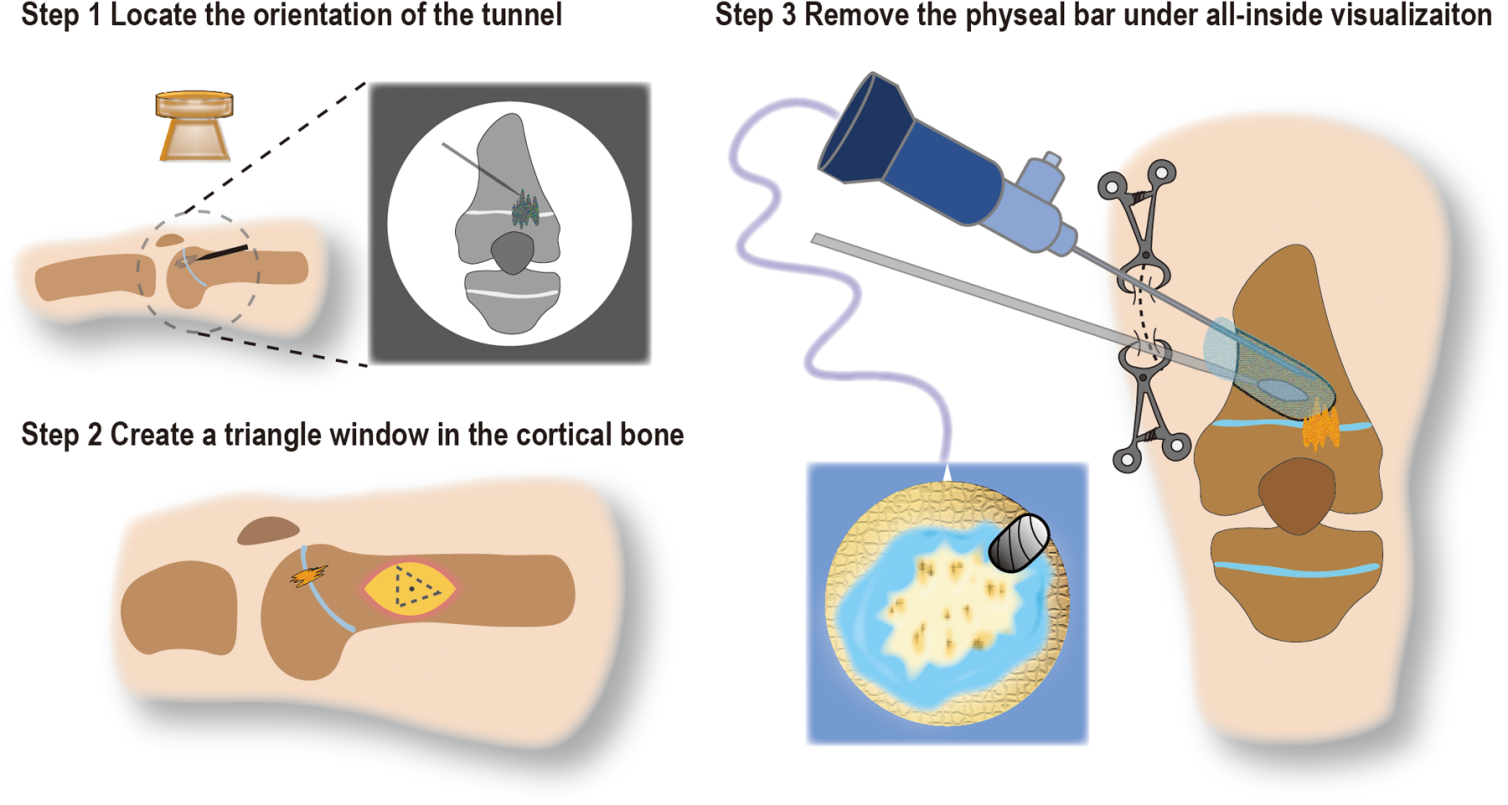
Schematic diagram of operation steps. Step 1 Use the K-wire to locate the orientation of the tunnel; Step 2 Based on step 1, create a triangle window in the lateral cortical bone (side length about 2.5 cm); Step 3 Temporally close the skin incision with towel clamps to form a closed osteocavity, remove the physeal bar under all-inside visualization of an arthroscope.

After general anesthesia, the patients were supine in the operating theatre with a tourniquet applied to the leg. The K-wire was used to locate tunnel orientation by fluoroscopy. Based on the preoperative planning and fluoroscopy, a skin incision was made to expose the metaphysis (Figure 1 Step 1). Then, a cortical triangle window (side length 2.5 cm) was resected with a predrilled screw hole (Figure 1 Step 2). Then, a 4 mm, 30° arthroscope was introduced into the established closed space, and a 2.9 mm burr was used to create a tunnel (Figure 1 Step 3). The skin incision was temporally closed by 2 or 3 towel clamps to keep the saline from leaking intraoperatively, and the saline bag could be raised up about 2.5 m high. It was essential to increase the water pressure and form a bloodless vision. In such conditions, it was clear to supervise the surgeon's operation. Under the direct all-inside visualization of the arthroscope combined with the K-wire, a tunnel was created based on the designed angle and length until the physis cartilage margin and physeal bar were identified. Next, the surgeon could quickly identify the physeal bar and remove all the physeal bars till a cartilage ring was formed. The depth size was generally 3–5 mm beyond the epiphyseal plate.

**Figure 2:**
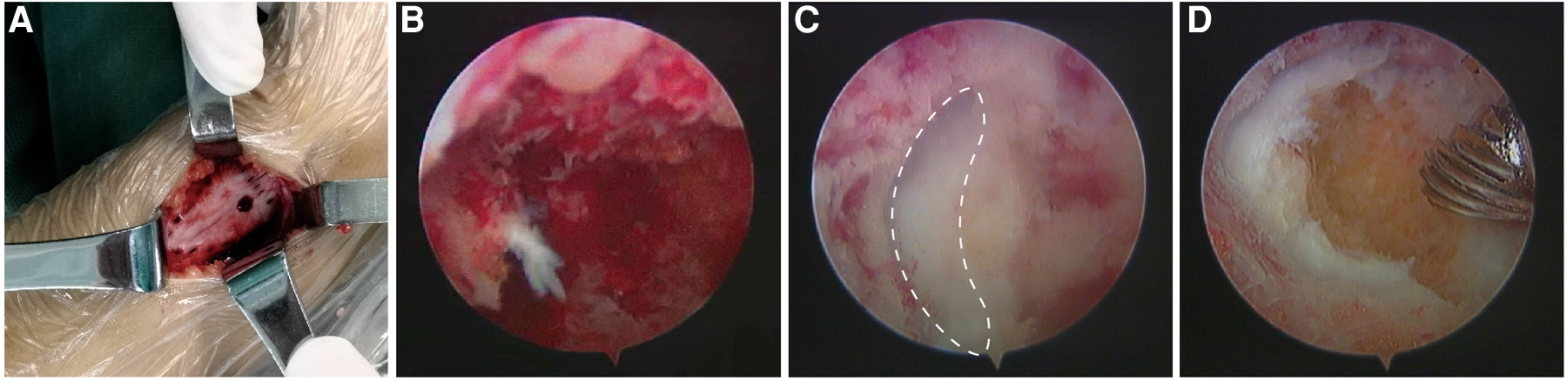
The surgical visualization of distal femur physeal bar resection. (**A**) A metaphyseal triangle window (side length 2.5 cm) was created with a screw hole predrilled; (**B**) Established a bone tunnel based on the designed tunnel orientation; (**C**) the bar was removed until the cartilage was encountered. The dotted line indicated the normal cartilage; (**D**) the bony bar was eradicated, and a physeal cartilage ring was identified.

After flushing with saline, bone wax was used to fill the entire bottom of the tunnel and forms a gourd-like structure, preventing the wax from shedding into the cavity as the limb grows. Care was taken to avoid damage to the periosteum. A 3.5 mm cortical screw was inserted into the predrilled hole to fix the cortical bone. The surgical procedure of a typical case of the distal femur physeal bar resection was shown (Figure 2, Supplementary Figure S2).

### Postoperative management

After surgery, a limb brace or plaster was applied for four weeks to avoid secondary fracture. Then, knee or ankle movement was permitted as tolerated by the patient. At postoperative week 8, the patient was allowed to place total weight on the leg. aLDFA or aLDTA, and Leg length discrepancy (LLD) were obtained to evaluate the effect of operation during follow-up.

## Results

In total, nine patients were included in this study. There were five boys and four girls. One patient received bilateral distal femur physeal bar resection. Five cases had previous fracture reduction and internal fixation operations, one had received conservative treatment for a fracture, and three had an unknown cause. The average age at the time of surgery was 8.3 ± 3.6 years old (ranging from 4.2 to 15 years old). The percentage of the bar area was 22.5 ± 7.7%. Five patients with the rapid development of varus or valgus deformity were treated more aggressively using epiphysiodesis simultaneously. No patient had an active infection at the time of surgery. The mean follow-up period was 28.5 ± 6.7 months. The demographic characteristics of the nine patients are shown in Table 1.

**Table 1:** Patient demographic and outcomes after surgical intervention.

Patient	Gender	Bar location	Bar type size (%)	Age at time of surgery (years)	Follow-up period (months)	Surgery time (h)	Epiphysiodesis simultaneously	Angular deformity (degree)^a^		LLD (cm)	
								Pre-op	Post-op	Pre-op	Post-op
1	Female	Distal femur (L)	Central, 26.0	6.6	45	3.5	No	26.7	4.4	1.4	1.1
2	Male	Distal femur	Mixed, 29.7	10.1	34	3.4	No	11.4	6.7	2.1	1.2
3	Male	Distal femur (L)	Central, 23.6	4.2	29	3	Yes	15.8	3.8	0.5	0.4
		Distal femur (R)	Mixed, 25.7	4.9	24	2.6	Yes	7.8	3.6	-	-
4	Female	Distal femur (R)	Central, 8.2	15	24	2.9	Yes	14.2	4.7	0.6	0.5
5	Male	Distal femur (L)	Central, 19.7	13	24	2.5	Yes	5.5	7.2	4.4	1.4
6	Male	Distal femur (L)	Central, 34.8	9.4	24	4.7	Yes	6.8	22.7	2.3	2.1
7	Female	Distal femeral (L)	Central, 24.9	8.7	26	3.2	No	26.7	13.4	0.6	0.3
8	Male	distal tibia	Central, 14.6	5.6	30	2.1	No	10.1	9.6	0.3	0.2
9	Female	distal tibia (L)	Mixed, 17.9	5.4	25	3.5	No	14.9	8.1	1	0.2

^a^ The angular deformity is the difference between the bilateral aLDFA or aLDTA. For case 3, 81° is used as a reference.

**Figure 3:**
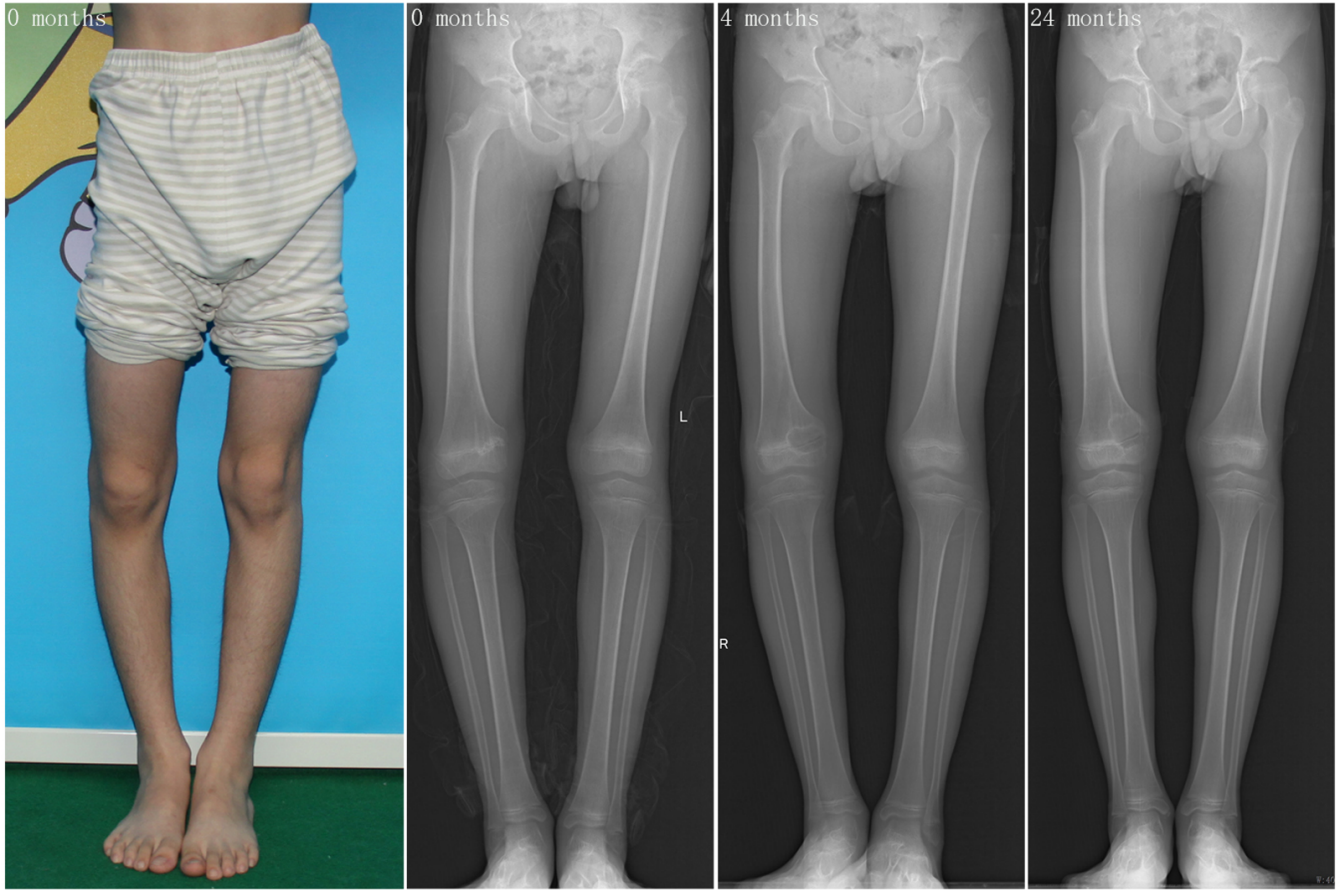
Ten years old boy who found progressively varus deformity of the right knee joint about one year (case 2): the gross view, anterior-posterior radiograph of the patient.

Before surgery, 6 (66.7%) patients had angular deformity >10 degrees. During the follow-up, eight (88.9%) patients with nine related joints had angular deformity improvement, averaging 8.3 ± 6.9 degrees (ranging from 0.5 to 22.3 degrees), and 1 (11.1%) patient had a worsening of the angular deformity. Before surgery, there were 5 (55.6%) patients with LLD >1 cm. All of the patients' affected limbs were shorter than the control ones. After surgery, we found that all 5 (55.6%) patients with LLD >1.0 cm had an LLD improvement, while 4 (55.6%) patients still had LLD >1.0 cm, ranging from 0.5 cm to 2.1 cm. The surgery time was 3.1 ± 0.7 h (ranging from 2.1 to 4.7 h). There were no postoperative fractures, infections, or intraoperative complications such as neurovascular injury. One of the patients was 15 at the age of surgery, while the skeletal age was 11, assessed by the elbow radiograph (Supplementary Figure S3) (15). The typical case is shown in Figure 3.

## Discussion

As partial physeal arrest alone could lead to angular and limb shortening deformity, physeal bar resection has been reported as an accepted treatment when less than 50% of the physis is damaged, more than two years of growth remains in the affected growth plate, and more than 2 cm predicted LLD (16). In this study, most of the patients could restore the growth ability of the physis after physeal bar resection. Only one patient had a worsening of the angular deformity >10 degrees. In this case, the physeal bar was scattered in the physis. During the physeal bar resection, removing all the physeal bars without physeal cartilage damage was difficult, and the surgery time significantly increased. The suitability of this type of bony bar for excision is questionable and needs further study.

Treatment of physeal arrest is a technically demanding procedure and not always successful, but critically necessary to reestablish growth in the extremity of a child who has sustained traumatic growth plate closure. Traditional surgical procedures are traumatic and prone to excessive removal of physeal cartilage or inadequate removal of bridging bone. The 3D fluoroscopy-based navigation system has increasingly been used in various fields of orthopedic surgery, including physeal bar resection (12, 17, 18). However, it is not widely used for the higher cost and more X-ray exposure. In 1981, Langenskiold described the use of the arthroscopy lamp as a light source but never reported the use of the arthroscope itself for direct visualization (6). In 1992, Stricker successfully used the arthroscope to assist in central bar removal in a single case of developmental growth arrest (7). Then, several scholars have reported the use of arthroscopically assisted resection of physeal bars (19–21). However, the vision for bar resection is bloody, and the surgeon can't remove the physeal bar under direct arthroscopic visualization. The arthroscope was just used for periodic imaging when the burr stopped working. It's not the ideal physeal bar resection under visualization. The main difference of this technique is that we create a closed osteocavity for the surgeon to resect and visualize the physeal bar simultaneously. It is all-inside visualization of the arthroscope. As far as we know, this study is the first successful attempt to use arthroscopic methods for physeal bar resection under all-inside visualization.

Due to the development of the arthroscopic instrument, arthroscopy surgery has been widely used in the past decade (22). It provides a clear surgical field to handle various complex situations in a closed natural joint space. During the physeal bar resection surgery, the most important reason to prevent the further use of an arthroscope is that the surgical area is small and open, which makes the physeal bar resection under direct visualization simultaneously impossible. Without a closed space, the fluid can't maintain a stable pressure environment which can stop bleeding from the wound. Besides, continued fluid irrigation can clear the surgical field in a closed area when blood obscures it, which is impossible in an open environment. Meantime, the smaller arthroscope has been used in the clinic. Hence, if a closed artificial osteocavity can be made, endoscopic resection of the physeal bar can become feasible. Inspired by arthroscopic surgery for soft tissue disease, we found that when the skin incision closed, a closed osteocavity was formed. In the beginning, we sutured the incision but found a lot of saline water leaked from the wound, and the effect of closing the cavity was not good. Then, the towel clamp was used, and the surgery field became clear. It was very convenient for the arthroscope to adjust the position to obtain maximum surgical area. Still, we should notify that this type of surgery has a certain learning curve, and the surgeon should be familiar with the use of arthroscope.

For severe deformity, physeal bar resection combined with guided growth has been reported as an alternative for angular deformity and LLD correction (3, 23). Mild deformity could be corrected after restoring partial growth arrest (16). Still, the indications for growth-guided surgery are controversial. Five patients received epiphysiodesis in this study, while one had a worsening angular deformity. There were no standard indications for the growth guide technique in this study. However, we think that the patients could be suggested the growth guide technique with one of the following criteria: (1) more than two years of skeletal growth remaining; (2) more than 10 degrees angular deformity; (3) the LLD >2 cm.

Anywhere there were some limitations in this study. First, the accuracy of this technique was not assessed by CT scan. Second, the sample size was small, and the follow-up period was limite.; We have no evidence that its surgical effect will eventually be better than the traditional methods. Other confirmatory studies with a more significant number of patients are warranted to validate the reliability of this modified technique.

## Data Availability

The raw data supporting the conclusions of this article will be made available by the authors, without undue reservation.
