# Supplementary figures and images for: Physeal bar resection by modified arthroscopically assisted surgery in a closed osteocavity
Source: Front Pediatr. 2023 Oct 17;11:1157192. doi: 10.3389/fped.2023.1157192 (PMC10616236; doi:10.3389/fped.2023.1157192)

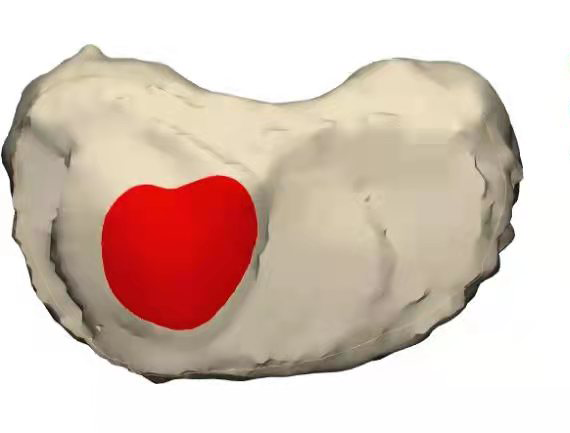

Supplement: Supplementary file 1 [file Image1.tif]

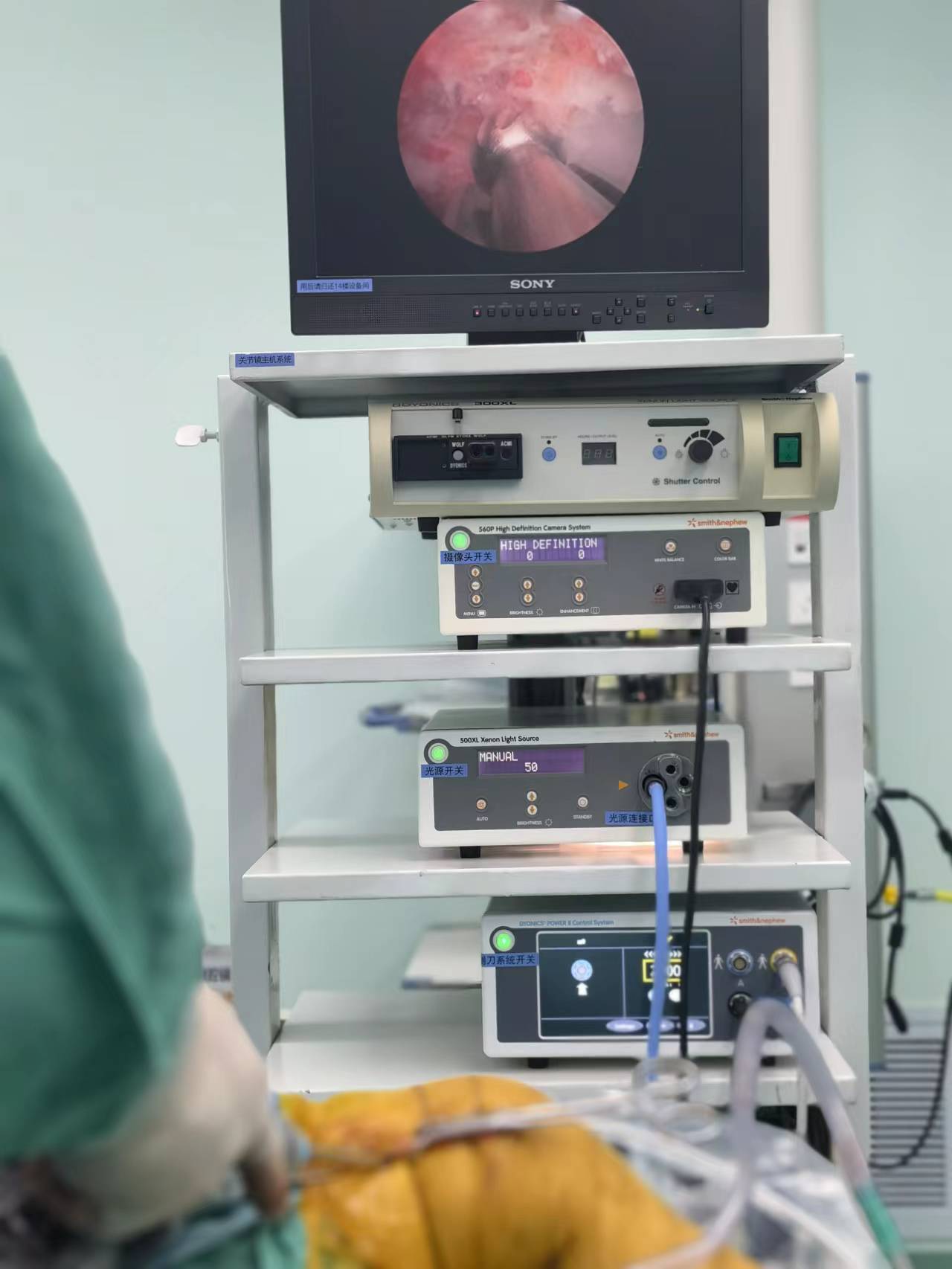

Supplement: Supplementary file 2 [file Image2.jpeg]

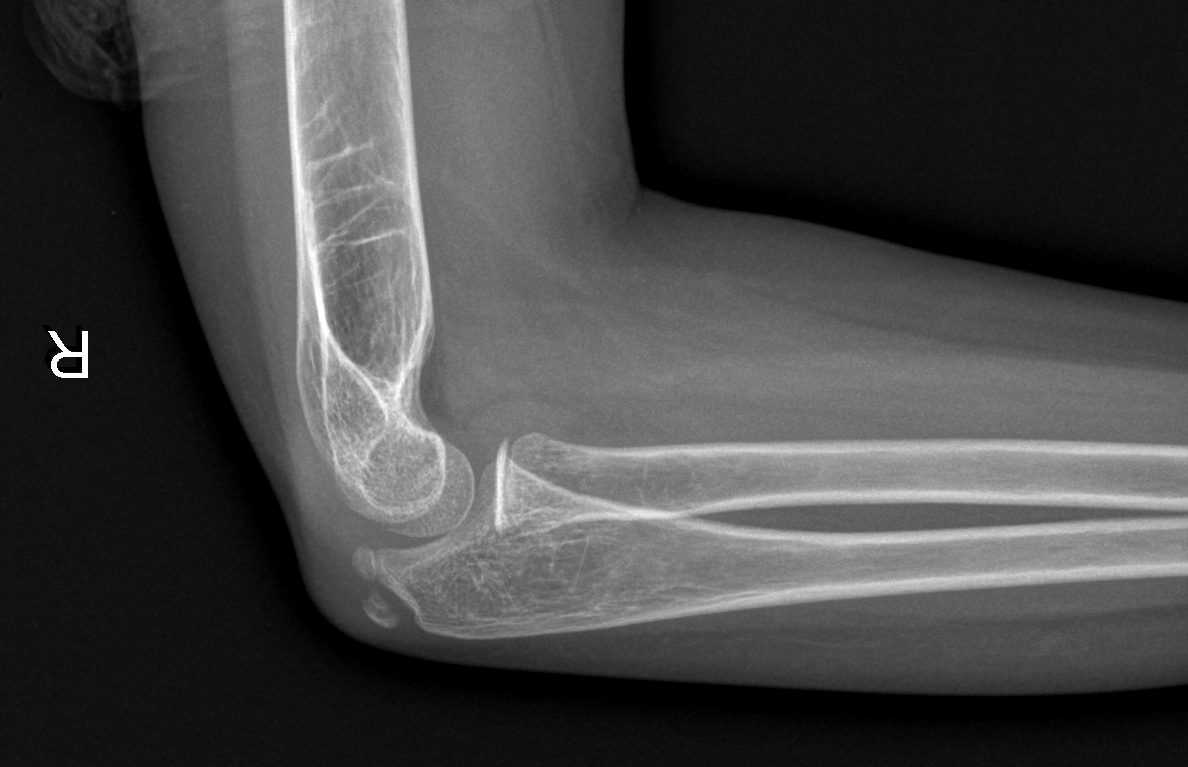

Supplement: Supplementary file 3 [file Image3.jpeg]
